# Afforestation driving long‐term surface water browning

**DOI:** 10.1111/gcb.14891

**Published:** 2019-11-29

**Authors:** Martin Škerlep, Eva Steiner, Anna‐Lena Axelsson, Emma S. Kritzberg

**Affiliations:** ^1^ Department of Biology Lund University Lund Sweden; ^2^ Karlskrona kommun Karlskrona Sweden; ^3^ Department of Forest Resource Management Swedish University of Agricultural Sciences Umeå Sweden

**Keywords:** afforestation, atmospheric deposition, browning, climate change, DOC, land use, water color

## Abstract

Increase in surface water color (browning), caused by rising dissolved organic carbon (DOC) and iron concentrations, has been widely reported and studied in the last couple of decades. This phenomenon has implications to aquatic ecosystem function and biogeochemical carbon cycling. While recovery from acidification and changes in climate‐related variables, such as precipitation and length of growing season, are recognized as drivers behind browning, land‐use change has received less attention. In this study, we include all of the above factors and aim to discern their individual and combined contribution to water color variation in an unprecedentedly long (1940–2016) and highly resolved dataset (~20 times per month), from a river in southern Sweden. Water color showed high seasonal variability and a marked long‐term increase, particularly in the latter half of the dataset (~1980). Short‐term and seasonal variations were best explained by precipitation, with temperature playing a secondary role. All explanatory variables (precipitation, temperature, S deposition, and land‐use change) contributed significantly and together predicted 75% of the long‐term variation in water color. Long‐term change was best explained by a pronounced increase in Norway spruce (*Picea abies *Karst) volume—a measure of land‐use change and a proxy for buildup of organic soil layers—and by change in atmospheric S deposition. When modeling water color with a combination of explanatory variables, Norway spruce showed the highest contribution to explaining long‐term variability. This study highlights the importance of considering land‐use change as a factor behind browning and combining multiple factors when making predictions in water color and DOC.

## Graphical abstract

The importance of considering land use (afforestation) as a factor in predicting water color (browning) and DOC concentrations in surface waters is highlighted in this study. While browning is likely caused by a combination of several factors (temperature, precipitation, S deposition, and land use), we here show the importance of considering land use, which was often not considered as an important factor in the past. When considering all factors together, 75% of variation in this long‐term water color data series can be explained, with afforestation being the most important predictor.

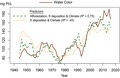

## 1 INTRODUCTION

Surface water browning, resulting from increasing concentrations of dissolved organic carbon (DOC) and iron (Fe), is frequently reported for northern freshwaters (Björnerås et al., 2017; Erlandsson et al., 2008; Kritzberg & Ekström, 2012; Monteith et al., 2007). This widespread phenomenon may alter greenhouse gas emissions from freshwaters and C storage in sediments (Heathcote, Anderson, Prairie, Engstrom, & del Giorgio, 2015; Lapierre, Guillemette, Berggren, & del Giorgio, 2013; Tranvik et al., 2009), affect the structure and function of limnic food webs (Creed et al., 2018; Solomon et al., 2015), enhance risks and costs associated with drinking water production (Matilainen, Vepsalainen, & Sillanpaa, 2010), and reduce the recreational value of lakes and running waters (Keeler et al., 2015).

Several factors appear to cause this browning (Bragee et al., 2015; Clark et al., 2010). Declining atmospheric sulfur (S) deposition and subsequent recovery from acidification has been shown to promote higher DOC concentrations by affecting solubility of organic matter and its lateral export from soils (Ekstrom et al., 2011; Evans et al., 2012). Changes in precipitation exert a direct control on the export of mobile solutes from soils to surface waters, and further modify water residence time, which is important in driving variation in water color, DOC, and Fe (de Wit et al., 2016; Kohler, Buffam, Laudon, & Bishop, 2008; Weyhenmeyer, Prairie, & Tranvik, 2014). Increasing temperatures promote decomposition and alter vegetation cover, making more DOC available for export from terrestrial systems (Finstad et al., 2016; Larsen, Andersen, & Hessen, 2011).

Recently, anthropogenic land‐use change has been suggested to play a significant role in the observed browning (Kritzberg, 2017; Meyer‐Jacob, Tolu, Bigler, Yang, & Bindler, 2015). In large regions of the northern hemisphere, forest cover has expanded at the expense of agricultural land, leading to increased buildup of soil carbon pools (Fang et al., 2014; Fuchs, Herold, Verburg, Clevers, & Eberle, 2015; Houghton, Hackler, & Lawrence, 1999). For instance, during the last century, southern Sweden has gone from an open largely agriculturally influenced landscape with dominance of grasses and deciduous vegetation, toward a landscape characterized by intensive forestry and a dominance of conifers, especially Norway spruce (*Picea abies* Karst; Lindbladh, Axelsson, Hultberg, Brunet, & Felton, 2014). Accumulation of soil organic carbon is higher in coniferous forests than in open land and deciduous forests (Hansson, Olsson, Olsson, Johansson, & Kleja, 2011) and the proportion of coniferous forest in the catchment is the best predictor of lake DOC concentration on spatial scales (Sobek, Tranvik, Prairie, Kortelainen, & Cole, 2007). Nevertheless, afforestation is rarely considered as an important factor driving browning over time.

While recovery from acidification, accelerating climate change, and anthropogenic land use all play a role in promoting browning, understanding their relative contribution on different temporal and geographic scales is essential for making future predictions.

It is unclear whether observed trends are on a trajectory toward a more “natural” state, as inferred from reduced acid deposition being the primary driver, or toward even browner water if forced by climate change. If anthropogenic land use is a major factor, management of the catchment may provide means to control browning. Disentangling the role of these factors to browning has until now been limited by a lack of datasets long enough to include large‐scale land‐use change, climatic variability, and the rise and decline in S deposition.

This study is based on water chemistry monitoring of Lyckeby River in southern Sweden, a dataset which is unique in its length (1940–2016) and sampling frequency (~20 measurements per month). The river has long been exploited for drinking water production, and has therefore been monitored for several physical and chemical parameters. At present, the catchment land cover is dominated by forest (>80%) and agricultural activity is limited to 6% of land cover. We complemented the water chemistry with data of relevant environmental variables, such as discharge, temperature, atmospheric S deposition, and total spruce volume in the catchment. Total spruce volume should reflect both areal coverage and stand age, and thereby correspond to the available soil organic matter pool (Rosenqvist, Kleja, & Johansson, 2010). With this dataset, we aimed to assess the environmental controls of short‐ and long‐term variability in water color. We hypothesized that temperature and discharge control short‐term changes (seasonal and periodical) in water color, while S deposition and afforestation (spruce volume) are the dominant drivers of long‐term browning. A previous study, using historic water color data from an adjacent region, hypothesized that the transition from agriculture to forestry was a major driver of browning of lakes in that area (Kritzberg, 2017). The dataset exploited in this study is stronger in terms of temporal coverage, and since land use is assessed by spruce volume rather than areal coverage, and therefore allows for more rigorous testing of this hypothesis and a stronger quantitative evaluation of confounding factors.

## 2 MATERIALS AND METHODS

### 2.1 Lyckeby River sampling site and monitoring data

Lyckeby River (approx. 100 km long) runs through counties Kronoberg, Kalmar, and Blekinge in southeastern Sweden. The total catchment area is 810 km^2^ and is dominated by forest (82%), followed by agricultural land (6%), urban areas (2%), and wetlands (2%). Surface waters represent 4% of the total catchment area. The main soil types in the catchment is glacial till (73%) and peat (8%; http://vattenwebb.smhi.se/modelarea/).

Lyckeby River has been utilized for drinking water since 1680, and currently provides drinking water to approximately 58,000 people. The water treatment facility in Lyckeby has kept continuous water chemistry and discharge data since 1940, providing a unique dataset for water color, organic matter, and discharge. All the mentioned variables have been measured every working day in the period 1940–2015 and twice a week after 2015. Averages of water color for July and August 1970 are missing from the time series. Water color measurements were made using a water color comparator based on comparing water color to hexachloroplatinate [PtCl_6_]^2−^ stock solution and are presented as mg Pt/L. For measuring organic carbon concentrations, chemical oxygen demand with KMnO_4_, according to Swedish standard 028118, was used until 2009. Since 2005, total organic carbon (TOC) concentrations were also analyzed by high energy combustion, according to Swedish standard (SS‐EN 1484).

### 2.2 Environmental data

Mean daily temperatures were obtained from the Swedish Meteorological and Hydrological institute (SMHI) and were measured 50 km from Lyckeby in Karlshamn. A number of growing degree days (GDD) were determined as number of days with mean temperature of 5°C or above.

Data on S deposition was based on deposition data from the European Monitoring and Evaluation Programme (grid 6161), processed as described (Moldan, Cosby, & Wright, 2013), and provided by the Swedish Environmental Research Institute IVL.

### 2.3 Norway spruce volume

Total volume of Norway spruce for the Lyckeby River catchment was estimated using data from the Swedish National Forest Inventory (NFI), which is a sample‐based inventory of forest resources designed to assess status and change at national and regional level (Fridman et al., 2014). We used a polygon layer from SMHI to extract NFI transects and plots surveyed in 1926 and between 1953 and 2016 within the Lyckeby River catchment from the NFI databases. From 1953 and onwards, calculations of total volume of Norway spruce within the watershed were performed as 5 year running means. The average error when estimating total volume of Norway spruce county of Blekinge is 14%, and the average error will be higher the smaller the area.

### 2.4 Statistical analysis

RStudio version R 3.4.3 (Kite‐Eating Tree) was used to perform all statistical analyses. The feasibility of using water color as a proxy for organic carbon was tested by correlating monthly water color with TOC and KMnO_4_ measurements (Pearson's correlation coefficient). Coefficients of variation were calculated for discharge and water color, to compare deviations from standard means in the time series. Breakpoint analysis was applied using the *breakpoints()* function with yearly mean water color, the suggested number of breakpoints was kept. To assess the correlation of water color with discharge and temperature on seasonal scales, the nonparametric Kendall rank correlation was used (Kendall's tau) for each individual period, as determined by breakpoint analysis. Monthly mean values for water color, discharge, and temperature were used. Moreover, residuals obtained from the correlation between water color and discharge were correlated against temperature, to see if the variation in the relationship between water color and discharge could be explained by temperature.

The significance of long‐term trends was tested by nonparametric Mann–Kendall trend test to yearly median values from 1940 to 2016. Absolute rates of change (e.g., mg Pt/L/year) were determined by the Theil slope of the Mann–Kendall trend test (Theil, 1950).

Locally weighted scatterplot smoothing (LOESS) fitting was applied in order to generate smooth trends of water color and explanatory variables. Yearly means for water color, discharge, temperature, and GDD were used, while only 5 year means were available for S deposition and Spruce volume. A span of 0.375 was used for LOESS smoothing of all the time series. To be able to better compare the different variables, *z*‐score (standard score) values were calculated. *z*‐Score is the number of standard deviations a data point is away from the mean of the dataset and is calculated as:

$$z=\frac{x-\bar{x}}{\sigma },$$

where *x *is the monthly mean, $\bar{x}$ is the variable mean for whole data series, and *σ *is the standard variation.

Partial least square (PLS) regression models were used to predict the variance in water color as a result of variation in the predicting variables. Explanatory variables in PLS analysis are ranked according to their relevance in explaining variation in the response variable, as variable importance in projection (VIP) values, where the most important variable for the model performance is assigned the highest VIP value (Wold, Sjostrom, & Eriksson, 2001). Explanatory variables with a VIP value ≥1.0 are considered important for the model performance (Wold, Trygg, Berglund, & Antti, 2001). The function *VIP()* in package plsVarSel was used to obtain VIP values. Additionally, standard coefficient values were calculated to quantify separate contributions of each variable in explaining water color variance in the PLS model.

Linear models using one or several explanatory variables, performed with the function *lm()* and the *predict()* function, were used to generate predicted values for water color using different combinations of explanatory variables in multiple linear regression (MLR; Table S1). When making linear models, 5 year running means were used for all variables to ensure comparability of data. Normality of residuals was verified with Q–Q plots and multicollinearity was assessed through variance inflation factors (VIF). Some collinearity was observed between factors temperature and GDD (VIF ≈ 7); however, models excluding one of the two factors did not increase the quality of the model. Akaike information criterion (AIC) values were calculated to assess the relative quality of the model. AIC estimates the quality of each model relative to other models; lower AIC values indicate a better model (Akaike, 1974).

## 3 RESULTS

**Figure 1:**
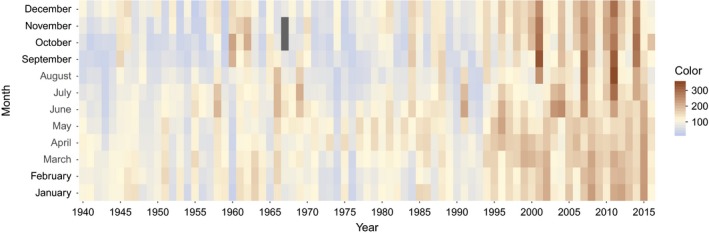
Monthly average water color (mg Pt/L) for the whole data series. Dark gray areas represent missing data

**Figure 2:**
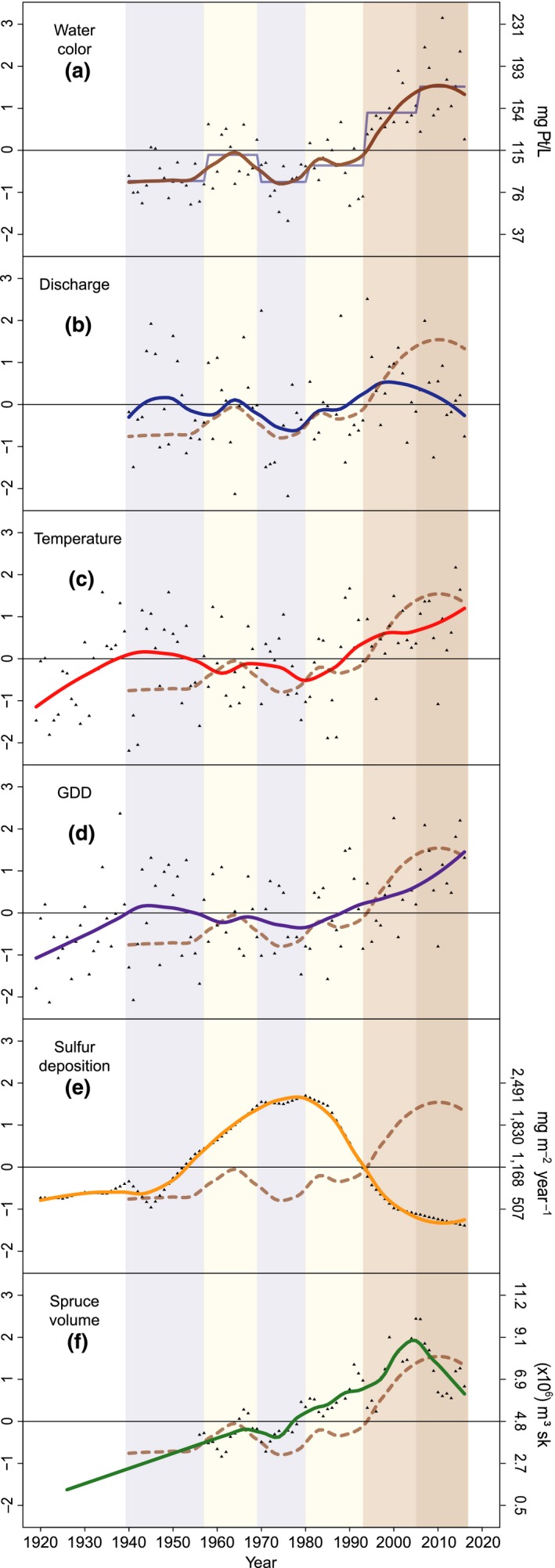
Variation in water color (a), discharge (b), temperature (c), GDD (d), S deposition (e), and spruce volume in catchment (f). Data is normalized as *z*‐scores (left *y*‐axis), with actual values provided on the right *y*‐axis of panels a, e, and f. The blue line in (a) shows break points for water color data, and the background colors correspond to these different periods (I–VI). Full smooth lines represent LOESS fits for each variable, while the dashed line in (b–f) represents the LOESS fits for water color

Water color was confirmed a good proxy for DOC by correlations with TOC (*r* = .95, *p* < .001, *n* = 134; 2005–2016; particulate carbon is generally <5% of TOC in these types of waters; Mattsson, Kortelainen, & Raike, 2005) and KMnO_4_ consumption (*r* = .88, *p* < .001, *n* = 805; 1941–2009). Monthly means of water color varied from 27 mg Pt/L (in January 1955) to 346 mg Pt/L (in October 2011) and averaged 115 mg Pt/L for the entire period (Figure 1). To assess periodicity in water color dynamics, we applied breakpoint analysis, which gave five breakpoints (1957, 1969, 1980, 1993, 2005; Figure 2a). Based on these, the data series was divided into six periods (I–VI). During period I, water color was relatively low, commonly below 100 mg Pt/L and on average 87 mg Pt/L. This was followed by a period of slightly higher color (II), reaching values around 200 mg Pt/L on several occasions and averaging 107 mg Pt/L. In period III, water color was again lower, on average 88 mg Pt/L. Color in period IV was commonly above 100 mg Pt/L and on average 103 mg Pt/L. During V and VI, water color was seldom below 100 mg Pt/L and commonly above 200 mg Pt/L, with an average of 143 and 172 mg Pt/L, respectively.

### 3.1 Seasonal variability

Water color was highly variable within years. In fact, the average coefficient of variation within years (29%) was similar to that of the variation between 1940 and 2016 (33%). Temperature in the region varied markedly between seasons, with minimum temperatures generally occurring in January (lowest monthly mean temperature −8.3°C in January 1942) and the highest in July (highest monthly mean temperature 20.5°C in August 2002). Discharge was also highly variable within years, that is, average coefficient of variation within years was 88%. High discharge periods were most frequent in winter or early spring (highest monthly discharge 31 m^3^/s in April 1951) and discharge was generally low between June and October (lowest monthly discharge 0.08 m^3^/s in October 1959).

Discharge affected seasonal variability in water color, as indicated by significant positive correlations for the separate periods in the time series (Table 1). Although water color showed significant correlations with temperature for some periods (I, III, IV), the direction of the correlation varied between the periods. Temperature did, however, explain some of the variation in the correlation between water color and discharge, that is, temperature correlated positively with the residuals from the correlation between flow and water color (Table 1). Thus, seasonal variability was clearly influenced by discharge and modulated by temperature.

**Table 1:** Kendall's tau correlations between water color and discharge (a), between water color and temperature (b), and between the residuals of water color + discharge and temperature (c)

Period	(a) Color/discharge	(b) Color/temperature	(c) Residuals/temperature
I (1940–1956)	0.43***	−0.17***	−0.15*
II (1957–1968)	0.23***	−0.05	0.15*
III (1969–1979)	0.16**	0.21***	0.08
IV (1980–1992)	0.13*	0.12*	0.25***
V (1993–2004)	0.21***	0.07	0.17**
VI (2005–2016)	0.31***	−0.05	0.17**

* *p* < .05;

** *p* < .01;

*** *p* < .001.

### 3.2 Long‐term trends and periodicity in water color and potential underlying drivers

Considering the entire period (1940–2016), water color increased by 84%, corresponding to 1.14 mg Pt/L per year (*p* < .001). This increase was, however, not linear over time, but displayed periodical dynamics, as demonstrated by the breakpoint analysis (Figure 2a).

By comparing the temporal dynamics of water color to those of environmental variables, the importance of potential drivers was explored. Periodicity in water color coincided well with the dynamics in discharge, especially in periods II–IV (Figure 2b), that is, the oscillations in the LOESS fits of water color and discharge were similar. However, the relatively high discharge in period I was not matched by high water color, and the high water color in period VI coincided with a period of low discharge. Unlike water color, discharge did not display a significant increase from 1940 until 2016 (*p* > .30). Finally, while linear regression with discharge as a single factor was significant, *R* ^2^ was relatively low (.26; *p* < .001).

There was little resemblance in periodical changes between water color and both yearly mean temperature and GGD (days above 5.0°C; Figure 2c,d). Both mean temperature and GDD, however, increased significantly during the period—0.015°C/year (*p* < .01) and 0.28 days/year (*p* < .001). Both temperature and GDD were on their own significant albeit not strong predictors of water color (*R* ^2^ = .26 and 0.38), and the relatively high temperature and GDD in the beginning of the time series were not matched by high water color.

For S deposition, a causal link would be reflected by a negative relationship. While the sharp decline in S deposition that started ~1980 (period IV) was paralleled by a general rise in water color, the increase in S deposition during periods I–III was not matched by a decline in water color (Figure 2e). Mann–Kendall analysis gave a significant decline in S deposition over the whole period (*p* < .01), and linear regression with S deposition as the predictor of water color was significant (*R* ^2^ = .43; *p* < .001).

The final variable included was total volume of Norway spruce in the river catchment. A considerable increase in spruce volume, by 508% or 58,600 m^3^/year, was estimated based on forest inventory records from 1926 to 2016 (Figure 2f). Linear regression with spruce volume as a single factor could explain 49% of the variation in water color. An apparent mismatch in the temporal development is the sharp drop in Spruce volume from 2005 where water color continued to rise.

**Figure 3:**
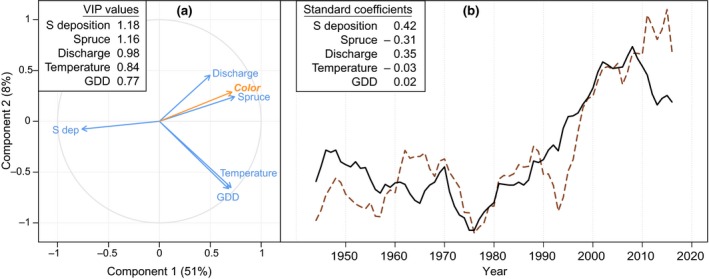
Correlation circle for partial least squares (PLS) regression (a) and predicted values based on PLS regression (b). Panel (a) illustrates correlations of the variables along the first two components of the PLS regression along with explained variation of water color for each component (in brackets). Panel (b) illustrates PLS predicted values (full black line) and actual water color (dashed brown line) based on 5 year running means. VIP values (a) represent variable importance in projection of each variable in the model and standard coefficients (b) represent the importance of each term in predicting variance in water color

To allow the influence of several environmental variables on water color, PLS and MLR analyses were applied. The PLS analysis could explain 59% of the variation in water color across two components, when all five predicting variables were included. Results from the PLS analysis show that S deposition and spruce volume were the two most influential factors in explaining water color variation, while discharge was also an important predictor (highest VIP values and standard coefficients; Figure 3). Spruce volume was positively correlated with water color indicated by a high positive standard coefficient (0.42). S deposition showed a negative correlation to water color (standard coefficient −0.31). Discharge also shows some correlation with water color based on the PLS analysis, with the VIP value close to 1 and positive standard coefficient (0.35). Temperature and GDD showed lower importance in explaining the variation of water color.

**Figure 4:**
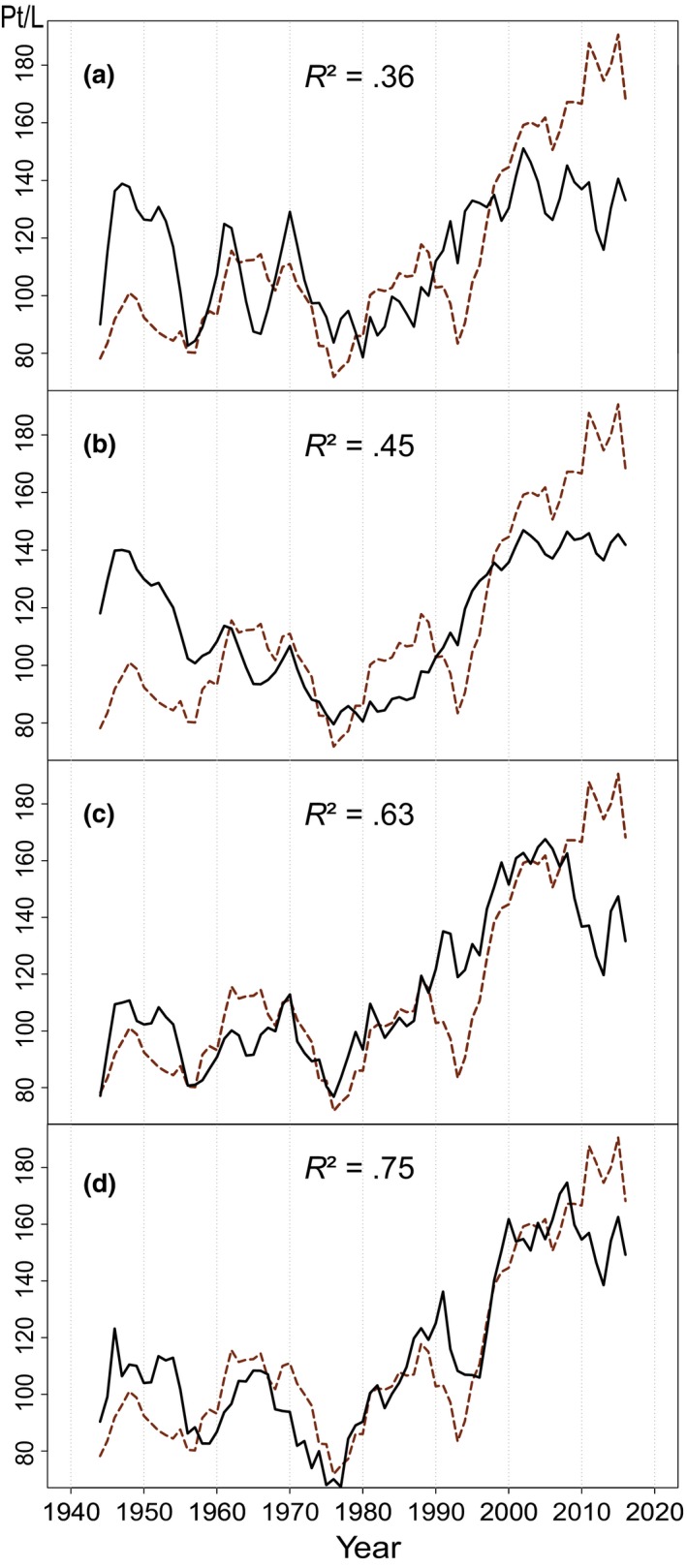
Measured water color (5 year running mean, dashed brown line) and predicted water color values based on multiple predicting factors (MLR, full black line); discharge and temperature (a), discharge, temperature and S deposition (b), discharge, temperature, and spruce volume (c), and discharge, temperature, growing degree days, S deposition, and spruce volume (d)

Including discharge and temperature in the MLR model explained 36% of the variation, compared to 26% for each variable separately, with both factors contributing significantly (Table S1; Figure 4a). Adding S deposition to the model made a significant contribution and resulted in an improvement in predictive power (45%). Including spruce volume in addition to discharge and temperature increased the predictability of water color even further to 63%. Allowing all variables, including GDD, the model explained 75% of water color variation. No model accurately caught the high values of water color measured in the end of the time series (Period VI, Figure 4). Notably, the models that did not include spruce volume also widely overestimated water color in the beginning of the time series.

## 4 DISCUSSION

Water color in Lyckeby River showed a strong increase over the 76 year time series, and temperature, precipitation, atmospheric S deposition, and land use all contributed significantly to this change in water chemistry. Previous studies have demonstrated that water color reflects DOC concentrations (Kritzberg, 2017), which was also supported in this study.

The main source of DOC are soil organic carbon pools in the catchment (Aitkenhead, Hope, & Billett, 1999; Weyhenmeyer et al., 2012), and precipitation plays an important role both in the production and transport of DOC from soil to surface waters (Haaland, Hongve, Laudon, Riise, & Vogt, 2010). The first‐order control that precipitation exerts on DOC export is supported by the seasonal covariation between water color and discharge, verified by correlations within the separate periods of the dataset. Water color also followed discharge on a periodical scale of a few years (Figure 2b). This positive relationship can reflect export rates from soils but also that soil wetness enhances microbial degradation and that shorter water residence times reduce processing of DOC within the aquatic flow path (Weyhenmeyer et al., 2012). However, there was no significant increase in discharge over the entire period, and discharge poorly predicted water color in the beginning and end of the data series. Thus, while lateral water flow is essential for connecting the catchment with the surface waters and plays a major role in seasonal and short‐term DOC patterns (Laudon et al., 2011), it cannot as a single factor explain the observed long‐term browning of this system.

Temperature played a secondary role to discharge in explaining seasonal water color fluctuations in this study. Temperature is correlated with seasonal variability in DOC exports to streams (Winterdahl et al., 2011), which may be related to regulation of organic matter decomposition, but also the constraint on lateral flow during soil frost periods (Laudon et al., 2012). On a longer timescale, increasing temperatures affect primary production, for instance by extending the duration of the growing season, thereby affecting the C cycle especially in temperature sensitive boreal regions (Larsen et al., 2011). Modeling water color from GDD gave a better fit than temperature, indicating that the length of the growing season may be an important factor for production and export of DOC in the catchment. Possibly, GDD as a variable may also capture the influence of warming on fluvial transport of DOC, which is limited under periods of frozen soils. Higher temperatures, promoting “greening” (increase in vegetation cover, primary production, litterfall, and exudates), have been shown to correspond to increasing DOC exports in Norwegian systems (Finstad et al., 2016). In the early part of the water color data series, however, both temperature and GDD were relatively high, while water color was low. In combination, discharge, temperature, and GDD predicted 51% of the water color variation in Lyckeby River, showing that climate variability and change may indeed play an important role in observed browning, but also suggesting that other factors must contribute.

Primary production and buildup of organic carbon pools, which constrain the quantity of DOC available for export, are also affected by land use and management. For instance, accumulation of soil organic carbon is higher in coniferous forest, than in open land, and intermediate in deciduous forest (Hansson et al., 2011), and accordingly soil water DOC is higher under coniferous than deciduous stands (Camino‐Serrano et al., 2014). In the present study, total volume of Norway spruce increased drastically, and this variable alone explained 49% of the variation in water color. When allowing the climate/weather factors (discharge, temperature, and GDD) to contribute, 63% of variation in water color could be explained. The high standard coefficient for spruce volume from the PLS further supports the importance of afforestation to browning of Lyckeby River. Paleolimnological studies show that early land use and catchment disturbance affected surface water DOC on a centennial timescale (Meyer‐Jacob et al., 2015), and Kritzberg (2017) suggested that browning of lakes in a region adjacent to this one is to a large extent driven by a landscape‐wide transition from extensive agriculture to forestry.

The use of tree volume in the current study integrates both areal extension of forest and tree growth, and should therefore reflect accumulation of organic matter better than catchment cover. Although values for total volume of Norway spruce between 1926 and 1957 were extrapolated, the assumption that there was a constant increase in spruce volume is supported by other studies on afforestation in the region (Fredh et al., 2012; Lindbladh et al., 2014).

Spruce volume also best corresponded with the low water color in the beginning of the time series. Given that less organic carbon had accumulated at this time, when tree stands were still young, there should have been less organic carbon available for degradation to DOC and lateral transport, which may explain why the high temperature and discharge during this period overestimate water color in the beginning of the time series. After 2005, there was an apparent mismatch between spruce volume and water color, coinciding with a large loss of forest biomass after a major storm (Gudrun). Even though forest biomass in southern Sweden was severely affected, the loss of tree volume did not necessarily correspond to a similarly large loss of soil organic carbon stocks. The fallen tree biomass was removed, but most of the organic carbon, which is the source of DOC, should have remained in the soil (Don et al., 2012). Thus, we believe that the poorer match between spruce volume and organic carbon in soils after Gudrun is the explanation for the poor prediction of water color toward the end of the time series. All together, the data suggest that including expansion of forest plantation and subsequent accumulation of organic carbon in soils is required to explain water color dynamics over time.

Sulfate deposition as a single factor, and in combination with discharge and temperature also offered high predictive power for water color (*R* ^2^ = .43 and .45, respectively), but failed to predict the low water color at the beginning of the time series. The sulfate hypothesis implies that water color declined as S deposition increased after around 1945 (WW2), as a result of increased acidity and ionic strength, which both suppress soil DOC mobility (Monteith et al., 2007). The present study area has experienced both high S deposition and has a bedrock with a rather poor buffering capacity, meaning acidification should be pronounced (Schopp, Posch, Mylona, & Johansson, 2003). However, little if any decline in water color was observed in response to increasing S deposition in Lyckeby River, as in another study based on historical data records (Kritzberg, 2017). This does not rule out that S deposition played an important role in DOC dynamics and browning, which is supported by several mechanistic and process focused studies (Ekstrom et al., 2011; Evans et al., 2012; Ledesma, Futter, Laudon, Evans, & Kohler, 2016), and is indicated here by the high VIP value for S deposition. Low pH and high ionic strength during peak deposition may have delayed the increase in water color by suppressing the mobility of DOC from the growing soil organic matter pool. Atmospheric S deposition during peak acidification has been high in southern Sweden compared to the rest of the country (Ferm et al., 2019), indicating that any effects of declining deposition should visible in the present study catchment. In relation to historic atmospheric S deposition, the area is comparable to average S deposition in the European Union (Engardt, Simpson, Schwikowski, & Granat, 2017) and northeastern United States, while deposition has been considerably lower in the western United States and Canada (Cheng & Zhang, 2017; Zhang et al., 2018).

This study supports the importance of land use as a factor behind long‐term browning. Afforestation and variability in climatic factors have acted in concert to increase the accumulation of organic carbon and the export to surface waters and could lead to further increases in water color. The rate at which water color increased in the past few decades may have been accelerated by increasing mobility of DOC in the wake of reduced S deposition, even if recovery from acidification alone was not sufficient to explain long‐term browning.

These conclusions are based on data from a region where spruce plantation has been extensive, which is the case for most of the hemiboreal and large parts of the boreal region in Sweden (Hedwall et al., 2019). Afforestation and reforestation have been widespread in Europe during the last century, ranging from the Baltic countries to the Mediterranean, particularly between the 1930s and 1980s (Fuchs, Herold, Verburg, & Clevers, 2013), and the boreal forest is an expanding biome in many regions, driven both by afforestation/reforestation and climate change (Settele et al., 2015). Stimulated also by atmospheric nitrogen deposition, increasing temperature, and longer growing seasons, it is likely to further promote an observed greening of the boreal region, that is, increasing primary production, biomass carbon pools, and tree lines moving upward (Finstad et al., 2016). Significant increases in forest density and volume are also observed in the North Central and Northeastern United States, since the 1950s, despite there being less change in forest area (Kauppi et al., 2006; Rautiainen, Wernick, Waggoner, Ausubel, & Kauppi, 2011). Furthermore, other land‐use management practices such as clear‐cutting, ditching, and peat burning have been shown to increase surface water DOC and could be important land‐use factors in DOC observed increases on a smaller scale (Clutterbuck & Yallop, 2010; Hribljan, Kane, Pypker, & Chimner, 2014; Yamashita, Kloeppel, Knoepp, Zausen, & Jaffe, 2011). Thus, while the importance of different drivers of browning varies in time and space, a reliance on data series beginning in the 1980s or 1990s has likely led to an underestimation of the role of land use in regions other than this one.

There has been an implicit assumption that changes in land use, such as afforestation, and consequent influence on water chemistry should be observed synchronously, when in fact the buildup of soil organic carbon stocks and DOC export progress slowly during several decades (Kritzberg, 2017; Rosenqvist et al., 2010). The link to forest management implies that browning may be possible to at least partially mitigate. As the role of land use and management has been largely overlooked as a factor behind browning, research has not been directed toward evaluating such measures. However, based on contemporary understanding of what factors govern the production and export of DOC in boreal landscapes, some options to mitigate browning were proposed and discussed by Kritzberg et al. (2019). Potential mitigation measures include actively promoting deciduous broadleaf tree species in riparian areas, and particularly so‐called discrete riparian input points, which have a disproportionally important influence on stream chemistry (Ploum, Leach, Kuglerová, & Laudon, 2018), manipulating groundwater levels, and increasing water residence time (Kritzberg et al., 2019). Future research efforts should evaluate how and where in the catchment altered management has potential to reduce export of DOC and thereby restrain browning. Moreover, future studies should explore if export of DOC continues to rise also centuries after coniferous forest is established, or if it stabilizes.
