# Supplementary material for: Afforestation driving long‐term surface water browning
Source: Glob Chang Biol. 2019 Nov 29;26(3):1390–9. doi: 10.1111/gcb.14891 (PMC7079054; doi:10.1111/gcb.14891)
Supplement: Supplementary file 1 [file GCB-26-1390-s001.docx]

Supporting information

**Table S1.** R^2^ values and Akaike information criterion (AIC) for different combinations of predicting variables, based on linear and multiple linear regression.

| **Predictor** | **R^2^** | **AIC** |
| --- | --- | --- |
| Discharge (D) | 0.26 | 696 |
| Temp. (T) | 0.26 | 696 |
| GDD (G) | 0.38 | 682 |
| S dep. (S) | 0.43 | 676 |
| Spruce (Sp) | 0.49 | 667 |
| D+T | 0.36 | 685 |
| D+S | 0.45 | 675 |
| D+Sp | 0.61 | 649 |
| D+G | 0.47 | 672 |
| D+T+S | 0.45 | 676 |
| D+T+Sp | 0.63 | 647 |
| D+T+G | 0.51 | 667 |
| D+T+S+Sp | 0.67 | 639 |
| D+T+S+G | 0.55 | 662 |
| D+T+Sp+G | 0.73 | 624 |
| D+T+S+Sp+G | 0.75 | 620 |
| D+S+Sp | 0.67 | 637 |
| D+S+Sp+G | 0.69 | 634 |
| D+S+G | 0.49 | 669 |
| D+Sp+G | 0.68 | 636 |
| T+S | 0.43 | 677 |
| T+Sp | 0.55 | 660 |
| T+G | 0.40 | 681 |
| T+S+Sp | 0.66 | 642 |
| T+S+G | 0.52 | 666 |
| T+Sp+G | 0.64 | 644 |
| T+S+Sp+G | 0.72 | 628 |
| S+Sp | 0.66 | 640 |
| S+G | 0.48 | 671 |
| S+Sp+G | 0.67 | 638 |
| Sp+G | 0.62 | 648 |

All models were significant (p<0.001). Lower AIC values indicate a better model.
